# Exploring pharmaceutical waste management in Southern Africa: a scoping review

**DOI:** 10.1080/20523211.2026.2700215

**Published:** 2026-07-28

**Authors:** Tessa Moleman, Aukje K. Mantel-Teeuwisse, Raffaella Ravinetto, Fatima Suleman, Jennie Lates, Saleh Aljadeeah

**Affiliations:** a Division of Pharmacoepidemiology and Clinical Pharmacology, Utrecht Institute for Pharmaceutical Sciences (UIPS), Utrecht University, Utrecht, The Netherlands; b Department of Public Health, Institute of Tropical Medicine, Antwerp, Belgium; c School of Public Health, University of The Western Cape, Cape Town, South Africa; d World Health Organization Collaborating Centre for Pharmaceutical Policy and Evidence-Based Practice, School of Health Sciences, University of KwaZulu-Natal, Durban; e Department of Pharmacy Practice and Policy, School of Pharmacy, University of Namibia, Windhoek, Namibia

**Keywords:** Pharmaceutical waste management, Southern Africa, environmental contamination, environmental health

## Abstract

Pharmaceutical waste management is an emerging health and environmental concern, particularly in low- and middle-income countries with limited waste infrastructure. This scoping review examined pharmaceutical waste practices and policies in Botswana, Eswatini, Lesotho, Namibia and South Africa at the healthcare facility and household levels. We systematically searched databases and grey literature sources from 2004 to 2024. Out of 729 research studies and 129 reports identified, we included 28 publications. The results were synthesised to show segregation, treatment and disposal practices. Regulatory frameworks existed in most countries, but often lacked detailed guidance and effective enforcement. Definitions of pharmaceutical waste varied, and documentation practices were inconsistent. Inappropriate practices of pharmaceutical waste management, such as inadequate incineration and landfilling conditions, were frequently reported. These findings are concerning and indicate a need for clearer, context-appropriate policies, stronger implementation mechanisms, improved segregation systems and further research, particularly at the household level.

## Introduction

Medicines are essential for the treatment, management and prevention of diseases, but they also contribute to waste, requiring careful management. Pharmaceutical waste is defined by the World Health Organization (WHO, 2025) as ‘Pharmaceutical products that are expired, unused, spilt or contaminated and prescribed and proprietary drugs, vaccines and sera that are no longer required and, due to their chemical or biological nature, need to be disposed of carefully’ (World Health Organization, 2025). Improper disposal of such waste can pose substantial risks to human health and the environment. For example, the release of pharmaceutical ingredients into water sources has been linked to the development of antibiotic-resistant bacteria and the disruption of aquatic ecosystems (Gwenzi et al., 2023). Yet, adequate disposal techniques are not always available, known or applied. For instance, in another low- and middle-income setting, a 2016 study of 195 health facilities in Nigeria found that 85% of pharmaceutical waste was disposed of by open burning, which may have exposed the operators and the environment to harmful pollutants in case of open burning (Obono, 2024).

Given the risks posed by pharmaceutical waste to both human health and the environment, it is essential to implement adequate waste management practices, including in resource-limited settings. The WHO has published many guidelines aimed at improving pharmaceutical waste management. The first one, issued in 1999 (World Health Organization, n.d.a), was followed by the WHO Pharmaceutical Waste Disposal Guideline of 2006 (World Health Organization, 2006), further updated in 2014 and 2025 (World Health Organization, 2014, 2025). The guidelines emphasise the importance of proper waste handling procedures. At a minimum, pharmaceutical waste should be segregated at the point of generation, treated to reduce its hazardous properties and disposed of using the safest and most environmentally sound methods that are locally feasible. Ultimately, the selection of disposal method depends on the type of pharmaceutical waste, and, unfortunately, the available resources.

Effective pharmaceutical waste management requires more than just selecting appropriate disposal methods; it also demands adequate resource allocation for the planning, implementation and monitoring of waste management systems at both the healthcare facility and household levels. As such, limited resources in low- and middle-income countries (LMICs) may hinder proper pharmaceutical waste management. Several studies have highlighted the absence or inadequate national guidelines for the handling of pharmaceutical waste in LMICs (Dom et al., 2023; Dori et al., 2024; Rogowska & Zimmermann, 2022; Sapkota & Pariatamby, 2023). Many countries in Africa lack guidelines and frameworks for healthcare waste management, let alone pharmaceutical waste management (Chisholm et al., 2021). Where guidelines do exist, they predominantly focus on waste generated in healthcare facilities, often neglecting the growing issue of pharmaceutical waste derived from households (Gwenzi et al., 2023; Rogowska & Zimmermann, 2022). The latter is also applicable to high-income countries: a scoping review of pharmaceutical waste practices globally found that 58.8% of unused or expired medicines from households are discarded as general waste, which means it ultimately ends up in municipal landfills, where they pose risks to the environment and public health (Dom et al., 2023).

While such global evidence highlights the scale of unsafe disposal practices, it does not fully capture the regional variations that influence how pharmaceutical waste is generated and managed. Existing evidence highlights the gaps in pharmaceutical waste management policies and practices in some parts of Africa, such as Eastern Africa, but there is currently no comprehensive study that examines the situation in Southern Africa (Karungamye et al., 2022). The Southern African region comprises countries with varying income levels and healthcare system capacities, which may influence pharmaceutical waste management practices. This study aims to map and present evidence from research studies and grey literature on the management policies and practices of pharmaceutical waste disposal in countries in the Southern African Customs Union.

## Methods

### Study design

This scoping review was conducted using the Arksey and O’Malley framework, updated by Levac et al. (2010). The five stages of this method are described below. The reporting of the results of this scoping review followed the PRISMA-ScR (Preferred Reporting Items for Systematic reviews and Meta-Analyses extension for Scoping Reviews) guidelines (Tricco et al., 2018). For this study, ‘Southern Africa’ is defined according to the UN sub-region definition, as well as the Southern African Customs Union (SACU), which includes Botswana, Eswatini, Lesotho, Namibia and South Africa. This definition was chosen because the SACU region represents a politically and economically integrated area (Development AD & SACU [Internet], n.d.). The review has been registered with the Open Science Framework (Exploring Pharmaceutical Waste Management in Southern Africa: A Scoping Review Protocol [Internet], 2024).

### Stage 1. Clarification of objectives and identifying the RQs

The overarching research question is: How is the disposal of expired or unwanted medicines, generated at the household and at healthcare facility level in Southern African countries, regulated and practiced? We aimed to answer this question by answering three more specific questions:

RQ1: What policies and regulations are in place for pharmaceutical waste disposal?

RQ2: What practices are currently used by governments, healthcare facilities, and households for the management of pharmaceutical waste disposal?

RQ3: What types and quantities of pharmaceutical waste are generated?

In this review, we focused on pharmaceutical waste as defined by the World Health Organization (n.d.b, 2014, 2025). For the scope of this review, we do not specifically address the additional procedures for antineoplastic waste (World Health Organization, 2014, 2017).

### Stage 2. Identifying relevant studies

We searched PubMed, Scopus and Web of Science databases from January 1, 2004, to November 31, 2024. Grey literature was retrieved from Overton and Google Scholar, as well as the United Nations Food and Agriculture Organization database (UN FAOLEX database), the WHO Institutional Repository for Information Sharing and the World Bank Group and SACU government institutions’ websites. Our search strategy was based on the Population, Intervention, Comparison and Outcome (PICO) framework. Search terms included combinations of keywords such as ‘pharmaceutical waste’, ‘drug disposal’, ‘healthcare facilities’ and ‘expired medicines’, as detailed in Appendix 1. Boolean operators ‘AND’ and ‘OR’ were used to structure the search. Reference manager software Zotero was used to identify and remove duplicate studies (Zotero [Windows 11], 2024). Snowballing was employed by examining the citations and reference lists of articles that fulfilled the inclusion criteria to identify additional relevant studies.

### Stage 3. Selection of relevant studies and reports

**Figure 1.**
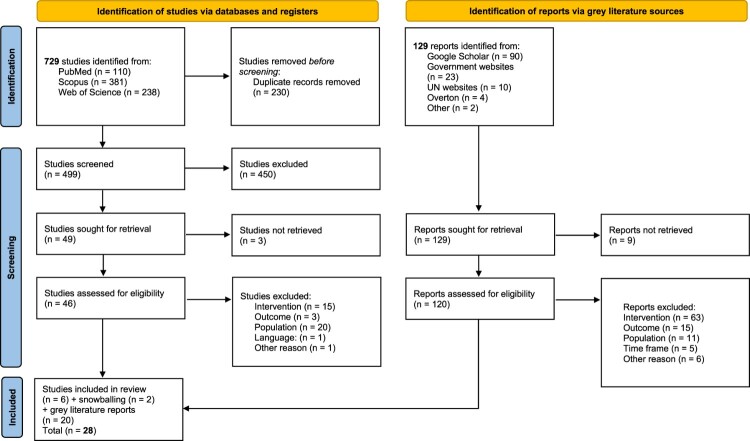
PRISMA flow chart of study selection. From Page et al. (2021).

We used Rayyan to screen the studies and reports yielded from our search over two phases (Ouzzani et al., 2016). The first phase involved screening of the title and abstract according to the inclusion and exclusion criteria presented in Supplemental Appendix 2. The second phase involved the full-text screening of all documents that passed the initial screening. This resulted in the final selection of studies and grey literature for inclusion in the review. Figure 1 shows the PRISMA flow diagram of the selection process.

### Stage 4. Extracting and charting the data

We extracted information from each included publication using a structured and iterative data extraction form in Microsoft Excel (Supplemental Appendix 3). The extraction form was refined during the process when it was determined that additional categories or fields were necessary to capture relevant information to answer our research questions.

### Stage 5. Collating, summarising and reporting the results

We summarised the results by country, noting the disposal and treatment techniques and existing policies reported per country in tabular form. The narrative synthesis focused on comparing common waste management practices and identifying barriers and opportunities for improvement across countries.

## Results

**Figure 2.**
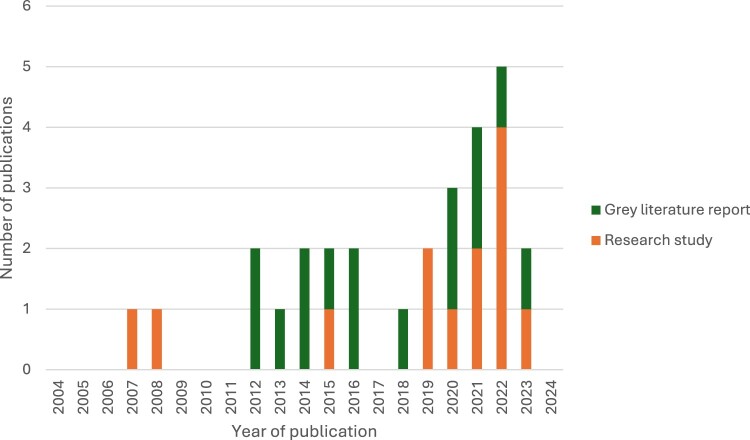
Distribution of publications about pharmaceutical waste management in the Southern African Customs Union over the years 2004–2024.

The database search yielded 729 studies, and the grey literature sources yielded 129 publications. After removing 230 duplicates, the screening of titles and abstracts of 499 studies resulted in the exclusion of 450 studies. We screened 166 full texts (46 research studies and 120 grey literature) against the pre-defined eligibility criteria. The full-text screening led to the inclusion of 6 research studies and 20 grey literature reports. Two additional reports were identified through snowballing, which resulted in the inclusion of a total of 28 studies and reports (Figure 1). Out of the 28 included publications, 26 were published between 2012 and 2023, with 2022 being the year with the most publications (Figure 2). Nine of the grey literature reports were master’s or doctoral theses. Most of the studies and reports focused on individual countries; only one publication covered multiple African countries (Chisholm et al., 2021). South Africa was the most represented country, with nine grey literature reports and eight research studies. Namibia, Botswana and Eswatini were represented by three, three and two publications, respectively. Only two grey literature reports were found for Lesotho.

Nineteen publications focused on waste from healthcare facilities, seven on household waste and two addressed waste from both healthcare facilities and households. Furthermore, only six of the publications focused solely on pharmaceutical waste. The other 22 publications addressed healthcare waste in general but included pharmaceutical waste within their scope.

### Defining pharmaceutical waste

Nineteen out of 28 sources explicitly defined what they meant by pharmaceutical waste. Twelve of these referred to ‘medicines’, whether expired, unused or spilled; while seven explicitly included packaging or materials contaminated by pharmaceuticals. For those that did not provide a definition, two sources provided examples, such as expired medicines or medicine containers, while seven left it undefined.

### Policies and regulatory frameworks

All SACU countries except Eswatini have established regulatory frameworks for healthcare waste.

The most significant policies and plans per country can be found in Supplemental Appendix 4. Botswana, Namibia and Lesotho incorporated pharmaceutical waste within broader national waste or healthcare waste management policies and supporting guidelines (Botswana Ministry of Health, UNICEF Botswana, 2022; Ministry of Health Government of the Kingdom of Lesotho, 2012; Republic of Namibia, 2011; World Bank Group, 2021). Eswatini relied primarily on its National Health Care Waste Management Guideline (Government of Eswatini, 2013). South Africa has the most advanced system, with national and provincial regulations, detailed standards and explicit requirements for medicine disposal and patient returns. However, even there, implementation varies by province (Chisholm et al., 2021; du Toit & Bodenstein, 2014; Maharaj et al., 2020; Motlatla, 2015; National Environmental Management: Waste Act: National Household Hazardous Waste Management Strategy | South African Government [Internet], n.d.; Olaniyi et al., 2019; South African Bureau of Standards, 2008).

### Waste segregation

We found 15 publications describing recommended and observed practices for pharmaceutical waste segregation within healthcare facilities; eight were from South Africa. An overview of the findings is presented in Table 1.

**Table 1.** Waste segregation recommendations and observed practices for each studied country.

Country	Recommended techniques	Observed practices	Source
Botswana	Brown labelled container or plastic bag	Mixed with infectious waste in red bags/containers	Botswana Ministry of Health, UNICEF Botswana (2022), Mmereki et al. (2017)
Eswatini	Green labelled container	–	Government of Eswatini (2013)
Namibia	Brown leak-proof container	Mixed with infectious waste in red bags/containers	Republic of Namibia (2011), Mushipe (2016)
Lesotho	Red plastic bag or container^a^	Expired medicines returned to supply hospital or collected in cardboard boxes	Ministry of Health Government of the Kingdom of Lesotho (2012), World Bank Group (2021)
South Africa	Different colours are recommended in different provinces	Mixed practices: Some studies report use of correct bins, others of mixed waste streams	Motlatla (2015), Ntloana (2021), Senekane and Masimula (2019), Masimula and Senekane (2020), Maseko (2014), Nemathaga et al. (2008), Msibi (2023), Motlatla and Maluleke (2021)

^a^ Lesotho does not have a separate container or bag for pharmaceutical waste. Instead, the waste is collected with infectious waste.

Although most countries, except for Lesotho, recommended segregating pharmaceutical waste using green or brown labelled containers or bags, actual practices reported in studies frequently diverged from these guidelines. Botswana recommended disposing of pharmaceutical waste in brown, labelled containers or plastic bags. However, studies found that expired medicines were often segregated in cardboard boxes or mixed with infectious waste (Botswana Ministry of Health, UNICEF Botswana, 2022; Mmereki et al., 2017). In Eswatini, green labelled containers were recommended for pharmaceutical waste (Government of Eswatini, 2013). No publications examined whether these recommendations were adhered to. In Namibia, publications reported different recommendations for the segregation of pharmaceutical waste. A 2011 national guideline differentiated pharmaceutical waste management by quantity, advising return of large quantities of expired medicines to hospital pharmacies, segregation of ward-level pharmaceutical waste at the point of generation and permitting disposal of small amounts of pharmaceutical or chemical waste with infectious healthcare waste (Republic of Namibia, 2011). A 2016 grey literature publication that reviewed practices in two hospitals noted the recommendation of brown labelled containers for pharmaceutical waste segregation, but observed that most of the pharmaceutical waste was mixed with infectious waste (Mushipe, 2016). Lesotho operates a three-bin system (red, black or yellow containers), which mixes pharmaceutical waste with infectious waste. It was reported that expired medicines are typically returned to local supply hospitals or stored in cardboard boxes at pharmacies (Ministry of Health Government of the Kingdom of Lesotho, 2012; World Bank Group, 2021).

Studies covering South Africa that recommended the use of green containers for pharmaceutical waste reported adherence ranged from 42.8% to 66.6% (Masimula & Senekane, 2020; Ntloana, 2021; Senekane & Masimula, 2019). Other studies described pharmaceutical waste as generally well segregated (Maseko, 2014; Motlatla, 2015). One study showed that knowledge of correct container use varied across professional groups, with pharmacists demonstrating the highest awareness and laboratory technicians the lowest in identifying the appropriate disposal container (Masimula & Senekane, 2020). However, two studies reported disposal of medicines in red plastic bags, which were sometimes also used to dispose of infectious waste (Msibi, 2023; Nemathaga et al., 2008).

### Treatment and disposal practices in healthcare facilities

Evidence for pharmaceutical waste management practices in healthcare facilities varied across countries. In some cases, descriptions of implemented practices were limited or unavailable, and national or institutional guidance documents constituted the primary source of information on how pharmaceutical waste is intended to be managed in practice. Consequently, this section synthesises both observed practices and formally recommended practices, which are presented together to reflect the best available evidence within each country.

Sixteen out of 28 publications specifically addressed recommended and/or observed practices for treating and disposing of pharmaceutical waste in healthcare facilities. Incineration and landfilling were the most common observed methods for pharmaceutical waste in healthcare facilities (Table 2).

**Table 2.** Pharmaceutical waste treatment and disposal techniques in healthcare facilities in Southern Africa.

Country	Study method classification	Disposal/Treatment methods	Publication year	Author
Botswana	Literature review	- Incineration (unspecified temperature), open dumping, landfilling.	2021	Chisholm et al. (2021)
	Report	- Incineration (unspecified temperature), return of medicines to central stores.	2022	Botswana Ministry of Health, UNICEF Botswana (2022)
	Mixed methods	- Incineration (incinerators were noted to not be operating according to international best practices), uncontrolled dumping in municipal solid waste sites, uncontrolled landfilling, disposal in sewage system.	2015	Mmereki et al. (2017)
Eswatini	Guideline	- Recommendations: incineration (temperatures between 800 and 1200°C), inertisation followed by municipal landfilling, or disposal at a controlled hazardous waste landfill.	2013	Government of Eswatini (2013)
Lesotho	Report/plan	- Observed: Incineration (unspecified temperature) followed by random disposal of residuals in proximity to the incinerators. - Observed: one hospital pharmacist reports that a system for the destruction of the donated pharmaceuticals is not in place as it is not recorded as stock.	2012	Ministry of Health Government of the Kingdom of Lesotho (2012)
	Report/plan	- Observed: open dumping, burning and incineration (incinerators do not always operate above 1200°C). - Recommendations: small quantities may be incinerated, encapsulated or safely buried together with infectious waste. Large quantities incinerated or encapsulated; dilution and sewer disposal for low-hazard liquids; return to supplier	2021	Kingdom of Lesotho World Bank Group (2021)
Namibia	Self-administered questionnaire	- No specific programme to safely manage hospital pharmaceutical waste at two teaching hospitals in Windhoek.	2022	Chadyiwa et al. (2022)
	Report/plan	- Observed: low-temperature incineration or pit burning is current practice. Recommendation: dispose non-hazardous pharmaceutical waste with municipal waste, return hazardous pharmaceutical waste for neutrialisation or incineration above 1200°C. If this is not possible, inertisation and disposal in hazardous waste landfill.	2011	Ministry of Health and Social Services Namibia Republic of Namibia (2011)
	Self-administered questionnaire	- Among respondents from the public hospital, 90.4% reported incineration as the method used for treating healthcare waste, while 5.8% reported steam autoclaving, 1.0% microwaving and 2.9% other treatment methods. - Respondents from the private hospital most frequently reported incineration (88.8%), followed by steam autoclaving (4.1%), microwaving (2.0%) and other treatment options (5.1%).	2016	Mushipe (2016)
South Africa	Literature review	- Incineration (unspecified temperature), open dumping, landfills.	2021	Chisholm et al. (2021)
	Mixed methods	- Waste is transported to the Chief Pharmacist at the Regional Hospital who usually apply for an approval to destroy it from the Provincial Government and can only release it to the waste management company for destruction after obtaining such approval. - Unclear from the report how the waste management company treats the waste.	2019	Olaniyi et al. (2019)
	Open-ended and close-ended structured questionnaires	- 3.4% of nurses and medical practitioners said that they return expired or damaged medicines and tablets to their supplier for disposal.	2019	Senekane and Masimula (2019)
	Semi-structured interviews	- Recommendation: combustion and land disposal. - Observed: waste disposal is outsourced to other companies; staff could not describe how the waste was treated and disposed of by these companies.	2014	Maseko (2014)
	Hospital case studies	- One hospital uses a locally made incinerator like a fireplace using coal as fuel, the other uses one with diesel as fuel and with old technology. In both cases, incinerator ash is openly dumped near incinerators. - At landfills, waste is burned instead of being compacted and covered by soil. Landfills are not very secure from animals such as dogs.	2007	Nemathaga et al. (2008)
	Self-administered questionnaire	- 20.5% Mushipe (2016) of HPs perceived flushing medicines down the toilet as a correct method of medicine disposal despite the majority being aware of waste disposal SOPs. - 31.9% Thornber et al. (2026) correctly answered incineration.	2021	Mahlaba et al. (2021)
	Hospital case studies	- Autoclaving.	2016	Heunis (2016)

HCPs, Health care professionals.

Across the five countries, incineration was the most frequently recommended method for treating pharmaceutical waste in healthcare facilities (Government of Eswatini, 2013; Mahlaba et al., 2021; Maseko, 2014; Republic of Namibia, 2011; World Bank Group, 2021). However, observed practices showed incinerators operating under unspecified or below recommended temperatures (Botswana Ministry of Health, UNICEF Botswana, 2022; Chisholm et al., 2021; Ministry of Health Government of the Kingdom of Lesotho, 2012; Mmereki et al., 2017; Nemathaga et al., 2008; Republic of Namibia, 2011; World Bank Group, 2021).

In Botswana, besides incineration, additional recorded practices included open dumping, uncontrolled landfilling, disposal in sewage systems or returning of medicines to central stores without further instructions after this step (Botswana Ministry of Health, UNICEF Botswana, 2022; Chisholm et al., 2021; Mmereki et al., 2017).

In Eswatini, available evidence was limited to one guideline recommending high-temperature incineration (800–1200°C), inertisation followed by municipal landfilling or disposal at controlled hazardous landfills (Government of Eswatini, 2013).

In Lesotho, the waste management plan lists incineration (≥800°C) as the preferred method for large quantities, particularly cytotoxic agents and antibiotics, with encapsulation as an alternative where incineration is unavailable. Small quantities may be disposed of together with infectious waste through incineration, encapsulation or safe burial; however, the plan notes that temperatures reached may be insufficient to fully destroy pharmaceutical compounds (World Bank Group, 2021). Observed practices included low-temperature incineration, open dumping and burning. Other recommended alternatives included safe burial, return to supplier and, for certain low-hazard liquids, sewer disposal (Ministry of Health Government of the Kingdom of Lesotho, 2012; World Bank Group, 2021).

In Namibia, although incineration above 1200°C was the preferred method, low-temperature operation of incinerators was reported in one publication (Republic of Namibia, 2011). Where incineration was not feasible, inertisation and controlled landfilling were recommended. One study reported the absence of structured hospital programmes, while another found that most healthcare worker respondents identified incineration as the primary treatment method (Chadyiwa et al., 2022; Mushipe, 2016).

In South Africa, reported practices included incineration, both controlled and uncontrolled landfilling, open dumping and autoclaving (Chisholm et al., 2021; Heunis, 2016; Mahlaba et al., 2021; Nemathaga et al., 2008). In some settings, pharmaceutical waste was transported to regional hospitals for approval prior to release to outsourced waste management companies, although downstream treatment processes were not clearly described. One study reported the use of locally constructed or older incinerators, with incinerator ash openly dumped nearby (Nemathaga et al., 2008). Landfills were sometimes described as open sites where waste was burned in the open air rather than compacted and covered (Chisholm et al., 2021; Nemathaga et al., 2008). Survey findings also indicated variability in staff knowledge, with some healthcare professionals identifying flushing as an acceptable disposal method, while others identified incineration as the correct approach (Mahlaba et al., 2021; Masimula & Senekane, 2020).

### Household treatment and disposal practices

Observed practices of household pharmaceutical waste disposal were discussed in eight publications from three countries. These practices included returning medicines to pharmacies (‘take-back’) or improper disposal in general trash or sewage systems (Table 3).

**Table 3.** Household pharmaceutical waste disposal practices in Southern Africa.

Country	Study method classification	Sample size (*N*)	Household disposal practices	Publication year	Author
Botswana	Mixed methods assessment of HCFs	37	- There is no standardised disposal practice. Home-based care facilitators either transport waste to the nearest healthcare facility, or local authorities/DHMTs directly collect from generation points.	2022	Botswana Ministry of Health, UNICEF Botswana (2022)
	Focus group discussions with HIV/AIDS primary caregivers	82	- Return to healthcare facilities, burying, backyard burning, disposal in council bins. - 73% (60 caregivers) mentioned knowledge of others burying healthcare wastes. - Incineration at clinics.	2008	Kang’ethe (2008)
Eswatini	Face-to-face interviews and questionnaires with households	329	- 54% dispose with solid waste, 39% burn in backyard pits. - 4% dispose in pit latrines; 2% in open pits near homestead.	2023	Welile et al. (2023)
South Africa	Structured questionnaires with patients	171	- Flushing down the toilet (25.7%), sink (5.8%), or disposal in municipal bins (23.9%). - 7.0% return to healthcare facilities.	2022	Mahlaba et al. (2022)
	Structured, self-administered questionnaires with patients	484	- 50.5% dispose expired medicines in general waste bins. - 33% flush down the toilet. - 8-9.4% return expired medicines to hospitals/pharmacies.	2020	Maharaj et al. (2020)
	Structured, self-administered questionnaires with households	117	- 63% mix medicines with household waste. - 11.3% flush medicines down the drain.	2022	Magagula et al. (2022)
	Online survey questionnaires with households	189	- Majority (45%) return waste to healthcare facilities; however, 34% dispose in general waste bins, which end up in landfills.	2021	Ngobeni (2021)
	Self-administered questionnaires, interviews, and observations with home-based caregivers	80	- 22 caregivers leave waste at the homestead. - 9 discard waste as they see fit. - 20 burn waste. - 8 take the waste to the nearest health clinic. - 9 flush or dispose in pit latrines.	2018	Zikhathile and Atagana (2018)

HCF, Health care facilities; DHMTs, District health management teams.

In Botswana, household pharmaceutical waste was commonly returned to health facilities and collected by local authorities, but some waste was inadequately disposed of through open burning or burial (Botswana Ministry of Health, UNICEF Botswana, 2022; Kang’ethe, 2008). For Eswatini, a study reported that disposal in the general municipal bins (54%) and burning in backyard pits (39%) were the most common practices (Welile et al., 2023). For South Africa, four studies noted that between 24 and 63% of the studied participants dispose of waste with the general municipal waste (Table 3) (Magagula et al., 2022; Maharaj et al., 2020; Mahlaba et al., 2022; Ngobeni, 2021). Additional reported practices included flushing medicines down the toilet or sink. Three out of five studies showed that only a small portion of respondents return medicines to healthcare facilities (Maharaj et al., 2020; Mahlaba et al., 2022; Zikhathile & Atagana, 2018), while one study reported return rates as high as 45% (Ngobeni, 2021). As the included studies were predominantly questionnaire-based and focused on self-reported disposal behaviours, downstream waste management processes beyond the point of return were not captured.

### Barriers to adequate pharmaceutical waste management

Twenty-two out of 28 publications highlighted that healthcare workers often lacked knowledge about safe pharmaceutical waste take-back or disposal practices, with many reporting that they were either not trained or poorly informed about regulations and proper waste handling techniques (Botswana Ministry of Health, UNICEF Botswana, 2022; Chadyiwa et al., 2022; Chisholm et al., 2021; du Toit & Bodenstein, 2014; Government of Eswatini, 2013; Kang’ethe, 2008; Maharaj et al., 2020; Mahlaba et al., 2022; Maseko, 2014; Ministry of Health Government of the Kingdom of Lesotho, 2012; Motlatla & Maluleke, 2021; Msibi, 2023; Mushipe, 2016; Nemathaga et al., 2008; Ngobeni, 2021; Ntloana, 2021; Republic of Namibia, 2011; Senekane & Masimula, 2019; Welile et al., 2023; World Bank Group, 2021; Zikhathile & Atagana, 2018). Furthermore, 11 studies noted the absence of or limited implementation of national policies and institutional guidelines (Botswana Ministry of Health, UNICEF Botswana, 2022; Chisholm et al., 2021; Magagula et al., 2022; Maharaj et al., 2020; Mmereki et al., 2017; Ngobeni, 2021; Ntloana, 2021; Republic of Namibia, 2011; Welile et al., 2023; World Bank Group, 2021; Zikhathile & Atagana, 2018). Inadequate infrastructure and insufficient resources were also emphasised as barriers to proper waste management by 10 studies. This included the lack of appropriate disposal containers, functioning incinerators, and an insufficiently allocated budget to manage healthcare waste (Botswana Ministry of Health, UNICEF Botswana, 2022; Chisholm et al., 2021; Kang’ethe, 2008; Maseko, 2014; Ministry of Health Government of the Kingdom of Lesotho, 2012; Mmereki et al., 2017; Motlatla, 2015; Nemathaga et al., 2008; Republic of Namibia, 2011; World Bank Group, 2021).

### Types and quantities of pharmaceutical waste

Reliable data on the quantities of pharmaceutical waste generated by healthcare facilities or households in SACU countries are scarce. Only one study, from South Africa, recorded absolute quantities: over a 12-month period, three hospitals generated 195.77, 322, and 457 kg of pharmaceutical waste, respectively. Across the three hospitals, pharmaceutical waste accounted for between 0.12% and 0.34% of the total healthcare waste stream, and was therefore not quantified every month (Heunis, 2016). Some healthcare facilities reported producing little or no pharmaceutical waste, which was attributed to limited overstocking (Masimula & Senekane, 2020; Ministry of Health Government of the Kingdom of Lesotho, 2012). However, publications from Lesotho and Botswana also noted a lack of documentation practices, which complicated accurate quantification of pharmaceutical waste volumes (Ministry of Health Government of the Kingdom of Lesotho, 2012; Mmereki et al., 2017).

## Discussion

This review highlights gaps between recommended standards and observed pharmaceutical waste management practices across SACU countries. While national guidelines, either specific to pharmaceutical waste or not, existed in all countries, they often lacked the level of operational detail needed for effective implementation at the facility level, contributing to inconsistent and fragmented approaches. Pharmaceutical waste was frequently not segregated from other healthcare waste streams and was often disposed of alongside infectious or general medical waste. Incineration followed by landfilling were the most reported methods of pharmaceutical waste treatment; however, many incinerators operated under substandard conditions and did not meet recommended safety or emissions standards. Landfilling, often without any pre-treatment of the waste or carried out in uncontrolled landfills, remained a common practice. Many stakeholders were unaware of safe take-back disposal protocols or the risks posed by improper pharmaceutical waste handling. In some cases, there is a lack of clarity on what happens downstream, after initial take-back. Combined with inadequate infrastructure, insufficient funding and weak enforcement mechanisms, these factors create persistent barriers to effective pharmaceutical waste management across the region.

Another important barrier to effective pharmaceutical waste management is the absence of a clear and comprehensive definition. If the working definition is limited to expired medicines, other types of waste may be overlooked. The definition of the WHO guideline (2025) explicitly encompasses a wide range of pharmaceutical products and related (contaminated) materials. Adopting this definition would help ensure that all relevant forms of pharmaceutical waste are consistently recognised, documented and appropriately managed (World Health Organization, 2025).

In addition to adopting a clear definition of pharmaceutical waste, strong policy frameworks are essential for ensuring safe and consistent waste management practices. A national healthcare waste management policy signifies a country’s commitment to improving health care and pharmaceutical waste management, but it won’t succeed without robust regulations for monitoring, evaluation and enforcement. Even then, effective implementation requires more than regulation: adequate financial resources, infrastructure and technical capacity are essential to translate policy into practice and ensure that healthcare waste management guidelines are consistently and safely applied across all levels of the healthcare system (World Health Organization, 2014). This review found that when countries, such as South Africa and Lesotho, have established national healthcare waste management policies in place, the lack of detailed, practical guidelines has hindered effective implementation at the facility and municipal levels (Maseko, 2014; World Bank Group, 2021). Moreover, household pharmaceutical waste remains largely underrepresented in national policy documents, possibly due to its relatively smaller volume compared to other healthcare facility waste. However, as medicine use increases, it is essential to include household-generated pharmaceutical waste in national waste management strategies (Rogowska & Zimmermann, 2022). Without integrated policy approaches that include household-level waste, countries risk undermining broader environmental and public health goals.

When it comes to technical aspects of treatment and disposal practices, some findings are crucial for health systems and policymakers, because they indicate a high level of human and environmental potential harm. The 2025 WHO guidelines prioritise high-temperature incineration or coprocessing in cement kilns (World Health Organization, 2025). However, our findings indicate that many incinerators in the region operate at temperatures below 800°C, failing to meet recommended thresholds (Mushipe, 2016; Republic of Namibia, 2011). Adequate incineration is often unavailable due to high installation and operational costs and the need for skilled personnel (Chisholm et al., 2021; Mmereki et al., 2017; Motlatla, 2015; Senekane & Masimula, 2019). Additionally, open dumping remains a common and extremely dangerous disposal practice across several countries.

Appropriate disposal of waste starts with *proper segregation*. While returning pharmaceutical waste to pharmacies or manufacturers is recommended by WHO guidelines, this option is not universally feasible (World Health Organization, 2025). Pharmacies in many settings lack the information and capacity to segregate the returned pharmaceutical waste. Support mechanisms are needed to empower pharmacies to carry out this task adequately. As for households, flushing down toilets, throwing in household garbage or other habits are persisting unsafe practices, reported both in our study and elsewhere, such as in East Africa (Karungamye et al., 2022). Public education campaigns may help to support responsible take-back practices at household levels, but they will only be effective if the next steps (adequate segregation at the pharmacy level and adequate final disposal) are also in place.

Although countries like Botswana, Namibia and South Africa have developed guidelines promoting more advanced segregation systems, our findings indicate that, in practice, pharmaceutical waste is still frequently mixed at the health facility level with infectious waste (Botswana Ministry of Health, UNICEF Botswana, 2022; Mmereki et al., 2017; Msibi, 2023; Mushipe, 2016; Nemathaga et al., 2008). In such cases, it is typically subjected to treatment methods intended for the latter, such as autoclaving or other steam-based disinfection, which are insufficient to neutralise all hazardous active ingredients, leaving the treated waste potentially harmful to the environment (World Health Organization, n.d.a, 2014, 2017, 2025).

Noteworthy, our findings concern pharmaceutical waste from human healthcare. Medicines for veterinary use are separately regulated, overseen and managed and little is known about waste management practices. However, the impact of poor practices is comparable, and there is a need to address it both in guidelines and regulations, as well as in research (Chisholm et al., 2021; Karungamye et al., 2022; Mmereki et al., 2017; Motlatla, 2015; Mushipe, 2016; Ngobeni, 2021; Republic of Namibia, 2011; Senekane & Masimula, 2019; World Health Organization, 2014).

The limited attention to pharmaceutical waste management in public health research in the SACU countries is consistent with the broader literature (Ravinetto et al., 2025). The management and final disposal of pharmaceutical waste continues to receive relatively little priority among national and international policymakers (Ravinetto et al., 2025). In many settings, the institutions responsible for the management and monitoring of pharmaceutical waste remain poorly defined. Defining the responsible authority for establishing regulations and standards for pharmaceutical waste management, identifying context-appropriate and feasible disposal interventions, monitoring these practices and enforcing compliance is an essential step towards improving pharmaceutical waste management. These responsibilities should fall within the remit of governments to protect both human health and the environment from the harmful effects of improper pharmaceutical waste disposal.

The countries included in this work are relatively stable and have well-developed pharmaceutical systems; South Africa, in particular, has a regulatory authority operating at a maturity level 3 as per the WHO Global Benchmarking Tool for vaccines (World Health Organization, 2026). It can be hypothesised that countries with fragile pharmaceutical governance, or characterised by extreme poverty and instability, would have a lower capacity to implement adequate guidelines, resulting in higher risks to environmental and public health. We believe that Botswana, Eswatini, Lesotho, Namibia and South Africa represent a valuable case in point, and that the recommendations that emerged here can be adapted and adopted in other LMICs. This approach is also applicable in different contexts. For instance, Thornber and colleagues recently applied a systems-based approach to develop transformational national mitigation strategies for a UK case study, hoping that it will support other high-income countries in minimising the public and environmental health risks associated with pharmaceutical pollution (Thornber et al., 2026).

### Strength and limitations

This scoping review has several strengths. First, it adopted and followed a systematic approach across the different stages of the review, which is in line with PRISMA-ScR guidelines. Secondly, by explicitly incorporating both healthcare facility and household pharmaceutical waste, the review addresses a critical, yet often overlooked, dimension of pharmaceutical waste management. This is the first study to examine pharmaceutical waste management practices in Southern African countries at this breadth and detail. Additionally, the countries included in this review represent a diversity of regulatory capacities and systemic contexts. Nonetheless, the review is not without limitations. The selection of countries was limited to a subset of Southern African countries. Several publications were not accessed due to paywalls, which may have limited our ability to capture the full scope of practices, challenges and contextual nuances. Additionally, the broad scope of the review across multiple countries may have obscured important subnational nuances, particularly in South Africa, where policies and practices can vary significantly by province. Future research could address this by conducting in-depth comparative studies across provinces or districts and evaluating how different legislative frameworks and levels of enforcement affect real-world pharmaceutical waste management practices. Finally, our review did not include cytotoxic medicines, which would be an interesting focus for future research given the specialised handling and disposal requirements involved, especially in resource-constrained settings.

## Conclusion

Our findings underscore the need for coherent, comprehensive and context-sensitive policies that clearly define pharmaceutical waste and outline practical disposal strategies. Inadequate definitions, weak implementation frameworks, limited infrastructure, and insufficient awareness contribute to inconsistent and often unsafe disposal practices at both the healthcare facility and household levels. Strengthening pharmaceutical waste management in the region, as well as in other LMICs with comparable gaps and capacities, will require clearer and more operational guidance aligned with WHO recommendations, improved segregation systems, sustainable and context-appropriate treatment options, greater investment in infrastructure, training and raising public awareness. These strategies must be backed by detailed policies, implementation guidelines, resources, infrastructure and technical capacities and training. This will require significant investments in infrastructure, public and professional education, and robust systems for monitoring evaluation, and enforcement. These investments are urgent, to protect human and environmental health from serious risks, including contamination of the soil and surface water, resulting in toxicity, contribution to antimicrobial resistance and unforeseen long-term effects on the flora, fauna and communities. Finally, given the lack of comprehensive data, future research should prioritise generating context-specific evidence to guide effective, equitable and environmentally sound pharmaceutical waste management strategies.

## Supplementary Material

Supplemental Material
