# Supplementary material for: Exploring pharmaceutical waste management in Southern Africa: a scoping review
Source: J Pharm Policy Pract. 2026 Jul 28;19(1):2700215. doi: 10.1080/20523211.2026.2700215 (PMC13421118; doi:10.1080/20523211.2026.2700215)
Supplement: Supplemental Material [file JPPP_A_2700215_SM5377.pdf]

Table of contents

Appendix 1: Search terms....................2

Appendix 2: Eligibility criteria....................4

Appendix 3: Data extraction form....................6

Appendix 4: Overview of the most important national policies and guidelines relating to pharmaceutical waste management for the studied Southern African countries....................8

Appendix 1: Search terms

Scopus database search string -18 November 2024

(TITLE-ABS-KEY ("healthcare facilities" OR "hospitals" OR "clinics" OR "medical centers" OR "pharmacy" OR "government agency" OR "public health authorities" OR "regulatory bodies" OR "households" OR "homes") AND TITLE-ABS-KEY ("management" OR "handling" OR "waste disposal" OR "medicine disposal" OR "disposal" OR "disposal systems" OR "incineration" OR "landfilling" OR "returns to manufacturers" OR "household practices" OR "knowledge" OR "practice" OR "policies" OR "regulations") AND TITLE-ABS-KEY ("Pharmaceutical waste" OR "medicine waste" OR "expired medicine" OR "unused medicine" OR "unwanted medicine")) AND PUBYEAR > 2003 AND PUBYEAR < 2025 AND (LIMIT-TO (LANGUAGE , "English"))

PubMed database search string - 13 November 2024

(("healthcare facilities" OR "hospitals" OR "clinics" OR "medical centers" OR "pharmacy" OR "government agency" OR "public health authorities" OR "regulatory bodies" OR "households" OR "homes") AND ("management" OR "handling" OR "waste disposal" OR "medicine disposal" OR "disposal" OR "disposal systems" OR "incineration" OR "landfilling" OR "returns to manufacturers" OR "household practices" OR "knowledge" OR "practice" OR "policies" OR "regulations")) AND ("Pharmaceutical waste" OR "medicine waste" OR "expired medicine" OR "unused medicine" OR "unwanted medicine")

(("healthcare facilities"[All Fields] OR "hospitals"[All Fields] OR "clinics"[All Fields] OR "medical centers"[All Fields] OR "pharmacy"[All Fields] OR "government agency"[All Fields] OR "public health authorities"[All Fields] OR "regulatory bodies"[All Fields] OR "households"[All Fields] OR "homes"[All Fields]) AND ("management"[All Fields] OR "handling"[All Fields] OR "waste disposal"[All Fields] OR "medicine disposal"[All Fields] OR "disposal"[All Fields] OR "disposal systems"[All Fields] OR "incineration"[All Fields] OR "landfilling"[All Fields] OR (("return"[All Fields] OR "returned"[All Fields] OR "returning"[All Fields] OR "returns"[All Fields]) AND ("manufacturability"[All Fields] OR "manufacturable"[All Fields] OR "manufacture"[All Fields] OR "manufacture s"[All Fields] OR "manufactured"[All Fields] OR "manufacturer"[All Fields] OR "manufacturer s"[All Fields] OR "manufacturers"[All Fields] OR "manufactures"[All Fields] OR "manufacturing"[All Fields])) OR "household practices"[All Fields] OR "knowledge"[All Fields] OR "practice"[All Fields] OR "policies"[All Fields] OR "regulations"[All Fields]) AND ("Pharmaceutical waste"[All Fields] OR "medicine waste"[All Fields] OR "expired medicine"[All Fields] OR "unused medicine"[All Fields] OR "unwanted medicine"[All Fields])) AND (2004/1/1:2024/11/13[pdat])

Web of Science database search 19 November 2024

Results for "healthcare facilities" OR "hospitals" OR "clinics" OR "medical centers" OR "pharmacy" OR "government agency" OR "public health authorities" OR "regulatory bodies" OR "households" OR "homes" (All Fields) AND management" OR "handling" OR "waste disposal" OR "medicine disposal" OR "disposal" OR "disposal systems" OR "incineration" OR "landfilling" OR "returns to manufacturers" OR "household practices" OR "knowledge" OR "practice"

OR "policies" OR "regulations" (All Fields) AND "Pharmaceutical waste" OR "medicine waste" OR "expired medicine" OR "unused medicine" OR "unwanted medicine" (All Fields) AND English (Language)

Appendix 2: Eligibility criteria

	**Criteria**	
**Domain**	**Inclusion criteria**	**Exclusion criteria**
**Time restriction**	(Grey) literature pubished between January 1, 2014 and 31 November, 2024.	Literature published before January 1, 2014.
**Publication type**	None	None.
**Language restriction**	Literature in English.	Literature in another language than English.
**Population**	Households, healthcare facilities, and government agencies within Southern African countries.	Households, healthcare facilities, and government agencies in countries other than those belonging to Southern Africa.
**Intervention**	Management of pharmaceutical waste, including methods, policies, and practices for disposing of expired or unwanted medicines, such as formal disposal systems (e.g., incineration, landfilling, returns to manufacturers) and informal or household practices.	Management of (healthcare) waste in general, without mentioning pharmaceutical waste.
**Outcome**	Pharmaceutical waste generated in healthcare facilities and households.	Environmental impact of pharmaceutical waste, pharmaceutical waste generated outside of healthcare facilities and households, publications that only focus on environmental impact and sustainability of waste methods.
**Setting**	Countries from the Southern African Customs Union, which includes Botswana, Eswatini, Lesotho, Namibia, and South Africa.	Non-Southern African Custom Union countries.

**Study type**	Reviews, review meta-analysis, qualitive studies, quantitative studies, mixed-methods studies, grey literature, UN agency reports, government reports, NGO reports.	None.

Appendix 3: Data extraction form

Main category	Subcategory	Description
**1. Author**		Name(s) of author(s).
**2. Year of publication**		Year the study/report/document was published.
**3. Title**		Full title of the study.
**4. Source (Journal/publisher)**		Name of the journal or publisher.
**5. Study objective**		The stated objective of the study.
**6. Study method classification**	a. Qualitative<br>b. Quantitative<br>c. Mixed-methods<br>d. Review<br>e. Report<br>f. Other/ not stated	
**7. Study design**		Describe methodology and methods.
**8. Country/countries or regions**		Country/countries that the study covers.
**9. Population description**		e.g. households, healthcare facilities, government agencies. Include eventual numbers.
**10. Disposal methods**		Disposal methods that are mentioned as most relevant in the study.
**11. Definition of pharmaceutical waste**		Working defenition that is used in the publication when refering to pharmaceutical waste.
**12. Types and quantities of pharmaceutical waste**		Which kind of pharmaceutical waste is mentioned, e.g. Solid, aquatic, specific drug types and what quantities.

**13. Policies, regulations and interventions in place to regulate medicine disposal**		Describe mention of any strategies in place to regulate medicine disposal and pharmaceutical waste.
**14. Challenges and barriers**		Key challenges noted in disposal practices and waste management (e.g. lack of infastructure, low awareness, limited government support).
**15. Knowledge gaps and future research**		Describe what knowlege gaps or recommendations for future research the study or report has identified.
**16. Other findings**		
**17. Notes**		Anything that stood out to the reviewer.

Appendix 1. Overview of the most important national policies and guidelines relating to pharmaceutical waste management for the studied Southern African countries.

Country	Year of publication	Issuing authority	Name of policy or guideline	Main emphasis
Botswana	1996	Department of Waste Management and Pollution Control	Clinical Waste Management Code of Practice	Provide information for proper healthcare waste management.
	2021	Ministry of Environment Natural Resources Conservation and Tourism	Integrated Waste Management Policy and Plan	Provide information on risks associated with health care waste, segregation of the waste, colour coding, as well as sections on the full continuum from generation, handling, transportation, treatment and final disposal.
Eswatini	2013	Ministry of Health	National Health Care Waste Management Guidelines	Provide practical information regarding waste management options.
Lesotho	2010	Ministry of Health	Healthcare Waste Management Policy	Support the implementation of the sustainable management of Health Care Waste from the health sector throughout Lesotho.
	2012	Ministry of Health	Consolidated Lesotho National Health Care Waste Management Plan for the Lesotho Maternal and Newborn Health Performance-Based Financing Project	Specific update of the National HCWM prepared in 2005. Provide the proper reference framework for the implementation of the sustainable management of Health Care Waste throughout Lesotho and in the context of the Lesotho Maternal and Newborn Health Performance-Based Financing Project.
	2016*	Ministry of Health	Infection Control and Waste Management Plan	Provide requirements for waste management of all types of waste from healthcare facilities in Lesotho.
Namibia	2010	Ministry of Health and Social Services	National Waste Management Policy	Provides information for collecting, transporting and incinerating sharps and other medical waste.

	2011	Ministry of Health and Social Services	Integrated Health Care Waste Management Plan	Provide the information to allow health care facilities to establish a good healthcare waste management system consistent with the regulatory requirements of Namibia.
South Africa	2016	Health Professions Council of South Africa	Guidelines for Management of Health Care Waste	Give criteria for the segregation, collection, movement, storage, and on-site disposal of waste materials within health care and biological research facilities.
	2024	Department of Forestry, Fisheries and the Environment	National Household Hazardous Waste Management Strategy	Recommendations for managing HHW and for municipalities to build on.

**Addendum of the plan in 2020 and 2021 for COVID-1*
